# The Association between Dietary Habits, Substance Use, and Mental Distress among Adults in Southern Norway: A Cross-Sectional Study among 28,047 Adults from the General Population

**DOI:** 10.3390/ijerph18189731

**Published:** 2021-09-15

**Authors:** Tonje Holte Stea, Linn Alvsvåg, Annette Løvheim Kleppang

**Affiliations:** 1 Department of Health and Nursing Science, University of Agder, 4604 Kristiansand, Norway; linn_alvsvag@hotmail.com (L.A.); annette.kleppang@uia.no (A.L.K.); 2 Department of Child and Adolescence Mental Health, Sørlandet Hospital, 4604 Kristiansand, Norway

**Keywords:** adults, mental distress, diet, smoking, smokeless tobacco, alcohol

## Abstract

The aim of the present study was to examine associations between dietary habits, substance use, and mental distress among adults. This cross-sectional study was conducted in 2019 using an online questionnaire and included 28,047 adults (≥18 years) from Southern Norway. Multivariable logistic regression models stratified by gender were used to examine the associations between different lifestyle behaviors and mental distress. The results showed increased odds of mental distress among males and females with low consumption of vegetables (OR:1.26; 95% CI:1.08–1.47 and 1.14; 1.02–1.28) and fish (1.28; 1.12–1.46 and 1.36; 1.22–1.52), and among females, but not males, with high consumption of sugar-sweetened beverages (1.25; 1.06–1.48) compared to those with a healthier consumption of these foods and beverages. The results also showed increased odds of mental distress among male and female smokers (1.38; 1.19–1.60 and 1.44; 1.26–1.64), and among females, but not males, reporting current use of smokeless tobacco (1.20; 1.03–1.40), compared to male and female non-smokers and female non-users of smokeless tobacco. Overall, unhealthy dietary habits, smoking and the use of smokeless tobacco was associated with increased odds of mental distress, but the relationship varied according to gender. Future studies are needed to confirm any possible causal relationships.

## 1. Introduction

The World Health Organization (WHO) has highlighted the need to prevent mental disorders, which have been identified as one of the major contributors to the global burden of disease [1,2]. In the OECD countries, up to one in five people are affected by a mental health condition at any time, and the direct and indirect economic costs of mental ill-health are estimated to be more than 4% of the GDP [3,4]. Research has shown a strong positive association between mental distress and mental disorders [5], and thus, identifying lifestyle behaviors that generate mental distress and may further develop into mental disorders is crucial.

Recent evidence has indicated that unhealthy dietary habits may increase the prevalence of mental distress, and that the relationship is bidirectional [6,7]. On the other hand, results from a cross-sectional study among Canadian adults indicated that high-quality diets and food security may prevent poor mental health [8], whereas poor diet quality did not predict mental distress among Dutch adults participating in a large cohort study [9]. Although few studies have examined the relationship between the consumption of specific food items and beverages, the results indicate that high consumption of fruits, vegetables, and fish, and low consumption of sugar-sweetened beverages may be associated with a reduced risk of developing mental distress and symptoms of depression [10,11,12].

Smoking has also been associated with an increased risk of poor mental health [13,14], and a longitudinal cohort study among U.S. adults showed that female tobacco users had an increased risk of mental distress compared to males [15]. Furthermore, studies indicate that the association between smoking and poor mental health appears to be bidirectional [16].

Even though smokeless tobacco is used across the globe and poses a major public health threat [17], there is a lack of studies examining the possible associations between the use of smokeless tobacco and mental distress. However, a cross-sectional study among U.S. adults found that the use of cigarettes and smokeless tobacco was more prevalent among individuals with mental distress [18].

Finally, studies have shown that long-term heavy alcohol consumption can increase the risk of mental distress and dependency [19,20], and the WHO described a causal relationship between the harmful use of alcohol and a range of mental and behavioral disorders [21]. Moreover, results have indicated that poor mental health may also be a maintaining factor for heavy alcohol consumption [22].

Few comprehensive studies previously examined the association between different lifestyle behaviors and mental distress, especially diet and smokeless tobacco. Thus, the aim of the present study was to examine the possible associations between diet, smoking, the use of smokeless tobacco, alcohol consumption, and mental distress among a large sample of adults living in Southern Norway.

## 2. Materials and Methods

### 2.1. Study Design and Population

**Figure 1:**
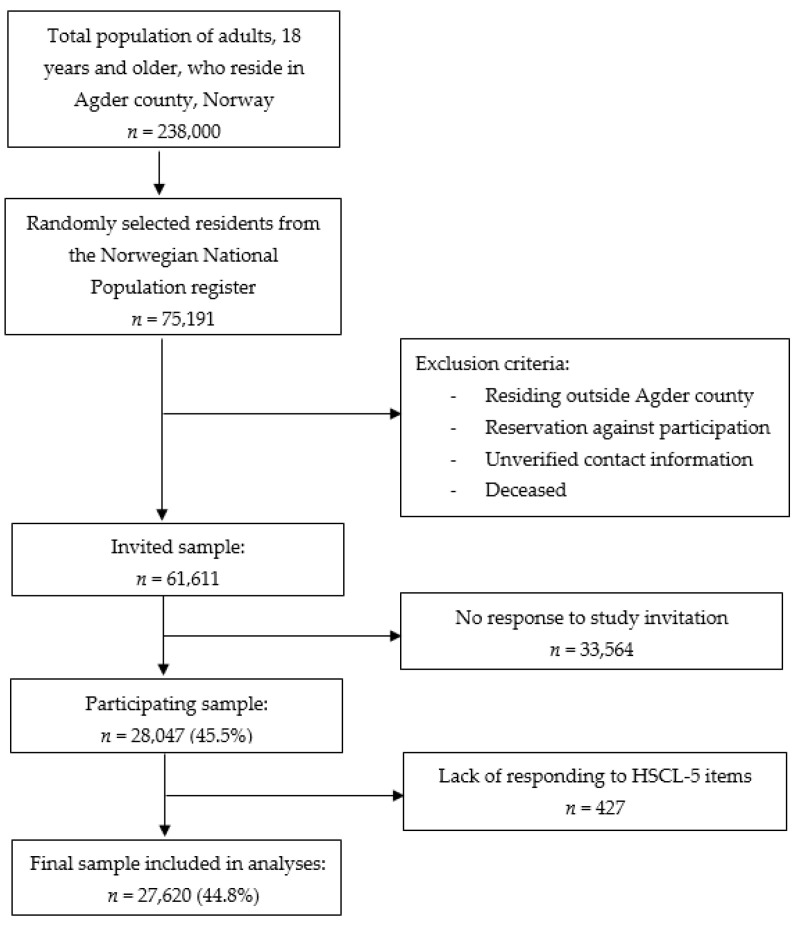
Sample collection flow chart.

The present cross-sectional study was part of the Norwegian Counties Public Health Surveys and was conducted between September and October 2019. A random sample of 75,191 adult residents (≥18 years of age) living in Southern Norway (31.6% of the adult population in this region) was drawn from the National Register. After the removal of deceased individuals, those registered in the Contact and Reservation Register with unverified contact information, and individuals registered with an address outside the Southern Norway region, a total of 61,611 residents (25.9%) were invited to participate. Invitations to participate were sent by e-mail and text message (SMS). Information about the study was broadcast through social media (Facebook, local webpages for the county and municipalities), regional and local newspapers, and television. To further increase the participation rate, six random participants each received a gift card worth NOK 4000 (approximately EUR 380). Participants gave their consent by filling out an online consent form and it took approximately 15 min to complete the online self-report questionnaire. In total, 28,047 individuals took part in the study (a response rate of 45.5%): 14,925 females (50.1%) and 13,122 males (41.2%) (Figure 1).

Participation in the present study was voluntary, and all participants had the opportunity to withdraw from the study at any time. The Norwegian Institute of Public Health held legal responsibility for the survey and was responsible for collecting and anonymizing data. Independent researchers who did not participate in the data collection process or had access to personally identifiable information analyzed data, and the study was conducted in line with the Declaration of Helsinki. Ethical approval and research clearance were obtained from the Faculty Ethical Committee at the University of Agder.

### 2.2. Measures

Questions, response alternatives, and variable definitions are presented in Table 1. Information about gender and age was retrieved from the Central Population Register.

Mental distress was measured using the short version (HSCL-5) of the Hopkins Symptom Checklist (HSCL-25) [23,24]. The HSCL-5 is acknowledged as a valid and reliable measure of anxiety and depressive symptoms among adults [24,25]. The instrument includes five items that refer to experiences during the past week, where feelings of nervousness or inner concern, fear or anxiousness, hopelessness about the future, unhappiness and restlessness are reported. Each item has the following four response alternatives: “not bothered” (coded 1), “a little bothered” (coded 2), “somewhat bothered” (coded 3), and “extremely bothered” (coded 4). A mean sum score was constructed by adding the number of each item divided by the number of questions. Further, the variable was dichotomized, and a validated cut-off score of >2.0 was used to identify participants with mental distress [25].

Diet and beverage consumption were assessed by asking respondents questions on how often they usually consumed fruit and berries, vegetables, and fish, and how often they usually drank sugar-sweetened beverages. The response alternatives were dichotomized into having high or low consumption of the selected food items (see Table 1).

Smoking and the use of smokeless tobacco were measured using a single question about how often participants smoked/used smokeless tobacco. The results were dichotomized to identify the current status as regards smoking habits and the use of smokeless tobacco.

High alcohol consumption was measured by asking respondents whether they had ever drunk alcohol or not. Response alternatives were “yes” and “no”. For responders who answered “yes”, the short version of the Alcohol Use Disorders Identification Test Consumption (AUDIT-C) was used to measure alcohol habits and potential alcohol problems [26]. A total AUDIT-C score between 0 and 12 points was calculated based on responses from three questions about alcohol use. In line with recommendations based on previous studies, a cut-off value of ≥5 for males and ≥4 for females was used to identify high alcohol consumption [27,28,29].

Information about perceived financial situation was used as a proxy for socioeconomic status. The response alternatives: “very difficult” and “difficult” were used to identify participants with perceived financial difficulties.

Body mass index (BMI) was based on respondents’ information concerning height in cm and weight in kg without clothes and shoes (kg/m^2^). Pregnant females were asked to report their weight before pregnancy. BMI was dichotomized to identify those who were overweight and obese (BMI ≥ 25) and those with a lower weight status (BMI < 25).

Information about age and gender was retrieved from the Central Population Register. Age was used as a continuous variable.

### 2.3. Statistical Analysis

Data were analyzed using SPSS (IBM Corp., Armonk, NY) version 25. Pearson chi-square tests were used to identify differences in mental distress between males and females with unhealthy dietary habits (Table 2) and substance users (Table 3).

In Table 4, multivariable logistic models adjusted for financial situation, BMI, and age were used to investigate the possible associations between low consumption of fruit and berries, vegetables, fish, high consumption of sugar-sweetened beverages, smoking, the use of smokeless tobacco, excessive alcohol consumption and mental distress (HSCL-5-score > 2.0). Separate models were presented for males and females. Pearson correlation tests revealed low pairwise correlations between independent variables in the models, indicating that multicollinearity was not present. The significance level was set to *p* < 0.05.

## 3. Results

Our sample included 27,620 participants: 46.8% males (*n* = 13,122) and 53.2% females (*n* = 14,925), 18 years of age and above (mean age 46.9 ± 16.3) in which 20.4% (*n* = 5547) reported having a difficult financial situation, and 79.6% (*n* = 21,696) reported no financial difficulties. Among all participants, a total of 3795 (13.7%) participants reported having mental distress, and the proportion was higher among females (15.3%) than males (11.9%).

The results presented in Table 2 show that, among participants with a low consumption of fruit and berries, and vegetables, a higher proportion of females than males reported mental distress (18% vs. 13%, respectively). Similarly, among individuals with a low consumption of fish and a high consumption of sugar-sweetened beverages, a higher proportion of females than males reported mental distress (24% vs. 19% and 29% vs. 19%, respectively).

The results presented in Table 3 show that among smokers, users of smokeless tobacco, and those who demonstrate excessive alcohol consumption, a higher proportion of females than males reported mental distress (22% vs. 19%, 28% vs. 16% and 19% vs. 14%, respectively).

Adjusted regression models (model 2 and 4, Table 4) showed increased odds of mental distress among males and females with a low consumption of vegetables (OR:1.26; 95% CI:1.08–1.47 and 1.14; 1.02–1.28) and fish (1.28; 1.12–1.46 and 1.36; 1.22–1.52), compared to those with a high consumption of vegetables and fish. Further, an increased probability of mental distress was shown among females, but not males, with a high consumption of sugar-sweetened beverages (1.25; 1.06–1.48), compared to those with a low consumption of sugar-sweetened beverages. The results also showed an increased probability of mental distress among male and female smokers (1.38; 1.19–1.60 and 1.44; 1.26–1.64), and among females, but not males, reporting current use of smokeless tobacco (1.20; 1.03–1.40), compared to male and female non-smokers and female non-users of smokeless tobacco.

The control variables indicated an increased probability of mental distress among males and females with financial difficulties (4.93; 4.36–5.57 and 3.59; 3.23–3.99) compared to males and females with no financial difficulties. Finally, our study revealed a decreased probability of having mental distress with older age among males and females (0.98; 0.98–0.98 and 0.97; 0.97–0.97) compared to younger participants.

## 4. Discussion

The results from the present study showed an association between unhealthy dietary habits, substance use, and mental distress in a large sample of Norwegian adults, but the relationships varied according to specific food items and beverages, different substance use habits, and gender.

Our findings indicated that low consumption of vegetables was associated with increased odds of mental distress, both among males and females. However, the observed association between low consumption of fruit and berries and mental distress seemed to disappear after adjustment for BMI, perceived financial situation, and age. A systematic review concluded that despite limitations resulting from the use of various methodologies in different population groups, the consumption of fruits and/or vegetables seems to have a positive influence on mental health [30]. Another recently published systematic review of cohort studies, however, reported that there is inconclusive evidence on the effect fruits and vegetables have on reducing the odds of developing depression and depressive symptoms [31]. An explanation for the possible positive effects of fruit, berries, and vegetables on mental health is the high content of polyphenols and antioxidants, which have been suggested to reduce oxidative stress and thereby have an alleviative effect on depressive symptoms [32,33]. Other studies also confirmed that the anti-inflammatory effect of diets rich in fruits and vegetables is causally linked to decreased risk of depressive symptoms [34,35,36].

Furthermore, results from the present study showed an association between a low consumption of fish and increased odds of mental distress in both males and females. Similar to our findings, other studies confirmed an association between a low consumption of fish and mental distress and symptoms of depression [37,38]. Moreover, a cross-sectional study among Spanish adults suggested a U-shaped relationship between the consumption of fish, the intake of long-chained omega-3 fatty acids, and depressive symptoms [39], whereas a meta-analysis of observational studies suggested a J-shaped association [37]. A recently published systematic review also suggested that omega-3 polyunsaturated fatty acids play an important role in the prevention and treatment of anxiety, and that the relationship may be explained by an anti-inflammatory response, brain-derived neurotropic factor (BDNF), cortisol, and cardiovascular activity [40].

Another result from the present study was the association between a high consumption of sugar-sweetened beverages and increased odds of mental distress among females, but not males. Various previous studies indicated a positive association between the consumption of sugar-sweetened beverages and mental distress in both genders [13,41], whereas a prospective study from the U.K. showed that high sugar consumption from sweet foods/beverages increased the chance of mood disorders in males but not females [42]. A study among females showed that exposure to negative emotions evoked by life problems increased energy intake, and that normal weight women only increased the consumption of sweet food, whereas overweight women increased the consumption of both sweet and salty foods [43]. Although most studies focus on the association between single foods or nutrients and mental health, it is likely that combinations of foods and the associated nutrients complement each other and have a synergistic effect on mental health status [44].

Further, results from the present study showed that smoking cigarettes was consistently associated with increased odds of mental distress in both males and females. Previous research suggested a bidirectional relationship between tobacco smoking and mental distress [17]; however, a systematic review concluded that the literature on the prospective associations between smoking, depression, and anxiety are inconsistent in terms of the direction of the most strongly supported association [45]. A cohort study among U.K. adults found no evidence to confirm an association between smoking and reduced symptoms of anxiety and stress [46], and a Mendelian randomization analysis of the Norwegian HUNT-study found no evidence of smoking leading to anxiety or depression [47]. Nevertheless, various studies confirmed that individuals suffering from depression and mental distress were more likely to be smokers as compared to those without symptoms of depression or mental distress, and that smoking cessation was associated with improved mental health [15,48,49]. Failure to quit smoking, on the other hand, has been shown to generate anxiety problems [46]. It is commonly believed that individuals smoke to self-medicate [45], and it is a matter of debate as to whether this theory is too frequently used as a justification for not restraining cigarette smoking, even though nicotine is not regarded as an appropriate therapy for any mental health problem [50].

The results from the present study also indicated an association between the use of smokeless tobacco and an increased probability of mental distress; however, for males, the association disappeared after adjustment for BMI, perceived financial difficulties, and age. Although few previous studies examine the possible associations between the use of smokeless tobacco and the prevalence of mental distress, a cross-sectional study among U.S. adults confirmed an increased use of cigarettes and smokeless tobacco among individuals with mental distress [19].

Unexpectedly, we did not identify an association between excessive alcohol consumption and mental distress, as the significant association observed among females disappeared after adjustment for BMI, perceived financial situation, and age. Evidence from other studies, however, confirmed an association between high alcohol consumption and the prevalence of mental distress among both genders [21,51,52]. A cross-sectional study among Korean adults concluded that the risk of mental distress was associated with the severity of alcohol-related problems and that the association appeared to be more evident among females than males [53]. Similar to cigarette smoking, consumption of alcohol has been identified as a common method of self-medication [54,55], and drinking to self-medicate anxiety and alleviate mood symptoms has further been associated with an increased risk of developing persistent alcohol dependence [56,57,58].

A limitation of the present study is its cross-sectional design, which precludes us from making any inferences about causal relationships. Furthermore, our study relies on self-reported measures. Thus, memory and recall biases and socially desirable responding may have affected our results. Another weakness of the present study is the limited participation rate, which must nevertheless be seen as acceptable compared to similar national and international studies among adults. Finally, our study only provided information about the association between the frequency of consumption of selected foods and beverages and mental distress. However, as the prevalence of depression and anxiety is associated with poor diet quality [59], we wanted to specifically focus on foods and beverages that have been identified as contributing to this, including a low consumption of fruit, berries, vegetables, and fish, and a high consumption of sugary beverages, which are the greatest source of added sugar.

A strength of the present study was the large sample of participants randomly drawn from a general population of adults. Moreover, we used validated and reliable tools to measure mental distress (HSCL-5) and high alcohol consumption (AUDIT-C) [24,60]. Furthermore, our results were controlled for BMI and age, in addition to perceived financial difficulties, which has been identified as a stronger and more robust predictor of mental distress compared to other commonly used objective measures of socioeconomic status [61,62].

## 5. Conclusions

The results from the present study confirmed that low consumption of vegetables and fish, and smoking was associated with increased odds of mental distress among both males and females. Moreover, high consumption of sugar-sweetened beverages and the use of smokeless tobacco were associated with increased odds of mental distress among females, but not males. No significant association was shown between excessive alcohol consumption and mental distress. Future longitudinal studies are needed to confirm a possible causal relationship between various lifestyle behaviors and mental distress, which may help practitioners to select appropriate interventions to prevent the development of mental disorders.

## Figures and Tables

**Table 1:** Questions, response alternatives, and variable definitions.

Questions	Response Alternatives	Variable Definitions
***Mental Distress***		
During the past week, to what degree have you been bothered with nervousness or inner concern?	Not bothered, a little bothered, somewhat bothered, extremely bothered	No mental distress (reference) Mental distress (HSCL-5 > 2.0)
During the past week, to what degree have you been bothered with fear or anxiousness?		
During the past week, to what degree have you been bothered with hopelessness about the future?		
During the past week, to what degree have you been bothered with unhappiness or sadness?		
During the past week, to what degree have you been bothered with concern or restlessness?		
***Diet and Beverage Consumption***		
How often do you eat fruits and berries (not including juices)?	Rarely/never, 1–3 times/month, once/week, 2–3 times/week, 4–6 times/week, daily	High consumption (reference) Low consumption (<6 times/week)
How often do you eat vegetables, included salads (not including potatoes)?		High consumption (reference) Low consumption (<6 times/week)
How often do you eat fish (as topping on bread, for lunch or dinner)?		High consumption (reference) Low consumption (<1 once/week)
How often do you drink sugar-sweetened soft drinks or beverages?		Low consumption (reference) High consumption (≥4 times/week).
***Tobacco***		
How often do you smoke? Including both filter cigarettes and rolling tobacco.	Daily, sometimes; not now, but daily in the past; not now, but sometimes in the past; have never smoked or used smokeless tobacco	Non-smoker (reference) Smoker (daily, sometimes)
How often do you use smokeless tobacco?		No current use (reference) Current use (daily, sometimes)
***Alcohol***		
Have you ever drunk any kind of alcoholic beverage?	Yes, No.	No excessive alcohol use (reference) Excessive alcohol use (score >5 for males, >4 for females)
Within the last 12 months, how often have you had a drink containing alcohol?	Never, ≤ once/month, 2–4 times </month, 2–3 times/week, ≥4 times/week	
How many drinks containing alcohol do you have on a typical day of drinking?	1–2, 3–4, 4–5, 5–6, 7–9, ≥10	
How often do you have six or more drinks on one occasion?	Never, < once/month, monthly, weekly, daily or almost daily	
***Perceived Financial Situation***		
If you live in a single-person household, think about your total income. If you live together with others, think of the total income of everyone in the household. How easy or difficult is it for you to make your money last on a daily basis, with this income?	Very difficult, difficult, relatively difficult, relatively easy, easy, very easy, don’t know	Easy financial situation (reference) Difficult financial situation (very difficult, difficult, relatively difficult)
***BMI***		
How tall are you without shoes? Answer in cm. How much do you weigh without clothes and shoes? Answer in kg. If pregnant, weight before pregnancy.		BMI < 25 (reference) BMI ≥ 25 (overweight/obesity)
***Age***		
Retrieved from the Central Population Register.	≥18 years old	Continuous variable (low, reference)

**Table 2:** Differences in mental distress between males and females with unhealthy dietary habits.

	Low Consumption of Fruit and Berries % (95% CI)			Low Consumption of Vegetables % (95% CI)			Low Consumption of Fish % (95% CI)			High Consumption of Sugar-Sweetened Beverages % (95% CI)		
	Males	Females	*p*-Value *	Males	Females	*p*-Value *	Males	Females	*p*-Value *	Males	Females	*p*-Value *
**Mental** **Distress**	13 (12–14)	18 (17–19)	<0.001	13 (12–14)	18 (17–19)	<0.001	19 (17–20)	24 (22–25)	<0.001	19 (17–21)	29 (26–31)	<0.001
**No Mental Distress**	87 (86–88)	82 (81–83)		87 (86–88)	82 (81–83)		81 (80–83)	76 (75–78)		81 (79–83)	71 (69–74)	

* χ^2^ tests were used to analyze differences in mental stress according to gender.

**Table 3:** Differences in mental distress between male and female substance users.

	Smoking % (95% CI)			Using Smokeless Tobacco % (95% CI)			Excessive Alcohol Consumption % (95% CI)		
	Males	Females	*p*-Value *	Males	Females	*p*-Value *	Males	Females	*p*-Value *
**Mental Distress**	19 (17–20)	22 (21–24)	0.004	16 (15–18)	28 (26–30)	<0.001	14 (13–15)	19 (18–20)	<0.001
**No Mental** **Distress**	81 (80–83)	78 (76–79)		84 (82–85)	72 (70–74)		86 (85–87)	81 (80–82)	

* χ^2^ tests were used to analyze differences in mental stress according to gender.

**Table 4:** Logistic regression describing the association between lifestyle behaviors and mental distress among adults.

		Model 1	Model 2	Model 3	Model 4
		Males, Unadjusted OR (95% CI)	Males OR (95% CI)	Females, Unadjusted OR (95% CI)	Females OR (95% CI)
**Exposure Variables**					
	***Fruit and berries***				
	High consumption	1	1	1	1
	Low consumption	1.27 (1.08–1.49) **	1.06 (0.89–1.26)	1.29 (1.16–1.45) ***	1.10 (0.97–1.24)
	***Vegetables***				
	High consumption	1	1	1	1
	Low consumption	1.19 (1.03–1.37) *	1.26 (1.08–1.47) **	1.18 (1.06–1.31) **	1.14 (1.02–1.28) *
	***Fish***				
	High consumption	1	1	1	1
	Low consumption	1.79 (1.59–2.01) ***	1.28 (1.12–1.46) ***	1.79 (1.62–1.98) ***	1.36 (1.22–1.52) ***
	***Sugar-sweetened beverages***				
	Low consumption	1	1	1	1
	High consumption	1.47 (1.27–1.69) **	1.13 (0.96–1.32)	1.81 (1.57–2.10) ***	1.25 (1.06–1.48) **
	***Smoke***				
	Not smoking	1	1	1	1
	Smoking	1.65 (1.45–1.88) ***	1.38 (1.19–1.60) ***	1.46 (1.30–1.65) ***	1.44 (1.26–1.64) ***
	***Smokeless tobacco***				
	Not using smokeless tobacco	1	1	1	1
	Using smokeless tobacco	1.30 (1.14–1.48) ***	1.01 (0.87–1.17)	1.83 (1.60–2.10) ***	1.20 (1.03–1.40) *
	***Alcohol***				
	Low alcohol consumption	1	1	1	1
	Excessive alcohol consumption	1.12 (1.00–1.25)	1.07 (0.94–1.21)	1.20 (1.08–1.32) ***	1.04 (0.93–1.16)
**Control Variables**	***BMI***				
	Normal weight		1		1
	Overweight/obese		1.02 (0.89–1.15)		1.06 (0.95–1.17)
	***Perceived financial situation***				
	Easy		1		1
	Difficult		4.93 (4.36–5.57) ***		3.59 (3.23–3.99) ***
	***Age***				
	Younger		1		1
	Older		0.98 (0.98–0.98) ***		0.97 (0.97–0.97) ***

*** *p* < 0.001; ** *p* < 0.01; * *p* < 0.05.

## Data Availability

Restrictions apply to the availability of these data. Data was obtained from Norwegian Institute of Public Health (NIPH) and are available at https://helsedata.no/en (accessed on 27 April 2021) with the permission of NIPH.
